# Machine learning-based prediction of hepatocellular carcinoma risk in steatotic liver disease: A nationwide cohort study

**DOI:** 10.1371/journal.pone.0349593

**Published:** 2026-05-28

**Authors:** Log Young Kim, Ji Soo Lee, Jeong-Ju Yoo, Eun Ju Cho, Sang Gyune Kim, Young Seok Kim

**Affiliations:** 1 Department of Big Data Strategy, National Health Insurance Service, Wonju, Republic of Korea; 2 Department of Internal Medicine, Division of Gastroenterology and Hepatology, Soonchunhyang University Bucheon Hospital, Gyeonggi-do, Republic of Korea; 3 Department of Internal Medicine and Liver Research Institute, Seoul National University College of Medicine, Seoul, Republic of Korea; Kaohsiung Medical University, TAIWAN

## Abstract

**Background and aims:**

Steatotic liver disease (SLD) has emerged as an important risk factor for hepatocellular carcinoma (HCC), often in the absence of cirrhosis. We aimed to develop explainable machine learning (ML) models to predict HCC risk in individuals with SLD using routinely collected screening data.

**Methods:**

Using the Korean National Health Insurance Service database, we included adults aged 20–79 years who underwent national health screening in 2017. SLD was defined as a fatty liver index (FLI) ≥ 60. Multiple ML algorithms, including deep learning models, were trained using a 7:3 train–test split with repeated non-replacement undersampling at a 1:3 case-to-control ratio to address extreme class imbalance.

**Results:**

Among 1,241,560 adults with SLD, 2,152 (0.17%) developed HCC during a 6-year follow-up period. In the internal validation cohort, the final weighted multi-head attention deep neural network ensemble achieved an area under the receiver operating characteristic curve of 0.923, with a sensitivity of 71.36% and specificity of 93.65%. SHapley Additive exPlanations consistently identified age, sex, triglycerides, total cholesterol, aminotransferases, gamma-glutamyl transferase (GGT), Charlson Comorbidity Index, and FLI as key contributors to HCC risk. In multivariable Cox models, older age, male sex, elevated GGT, higher aspartate aminotransferase and FLI, and greater comorbidity burden were positively associated with HCC risk, whereas higher triglyceride and total cholesterol levels were inversely associated. Model-based risk stratification identified four groups with distinct HCC-free survival curves; the extremely high-risk group had an approximately 74.9-fold higher hazard of HCC than the low-risk group (95% CI, 55.3–101.5).

**Conclusions:**

Overall, this explainable ML model based on routine health screening variables enables robust HCC risk stratification and may help inform future targeted surveillance strategies in SLD populations after external validation.

## Introduction

Steatotic liver disease (SLD) is the most common cause of chronic liver disease worldwide and is increasingly recognized as a major contributor to hepatocellular carcinoma (HCC) [1,2]. As the prevalence of metabolic syndrome rises, the global burden of HCC attributable to SLD continues to grow. Surveillance in this population is challenging because the annual incidence of HCC among SLD patients without cirrhosis is low; however, the large at-risk population results in a substantial absolute number of HCC cases [3,4]. Furthermore, a clinically meaningful proportion of HCC in SLD occurs in the absence of cirrhosis, limiting the effectiveness of surveillance strategies restricted to cirrhotic patients [5].

Current international guidelines generally advise against universal HCC surveillance in patients with SLD without cirrhosis due to insufficient evidence supporting its cost-effectiveness [6,7]. Consequently, most individuals with SLD are excluded from surveillance programs, even though high-risk metabolic phenotypes may go unrecognized in routine care. Conventional noninvasive fibrosis tests, such as FIB-4 or the NAFLD fibrosis score, serve as surrogates for fibrosis and predictors of HCC risk; however, their population-level application is limited by reliance on specialized testing and complete clinical data, which are often unavailable in unselected screening populations [8]. These gaps underscore the need for a precision screening method to identify the small subset of patients with SLD at high risk for HCC within the broader population.

Machine learning (ML) models can capture complex non-linear interactions among clinical variables and may improve risk discrimination under conditions of extreme class imbalance [9,10]. Using a nationwide cohort of more than 1.2 million adults with SLD, we developed and validated interpretable ML models to identify high-risk subgroups within the broader SLD population and to inform a targeted framework for HCC surveillance.

## Patients and methods

### Ethics statement

The study protocol was approved by the Institutional Review Board (IRB) of Soonchunhyang University Bucheon Hospital (IRB no. SCHBC 2025-04-006). The study was conducted in accordance with the Declaration of Helsinki and its subsequent amendments. The requirement for informed consent was waived by the IRB because the data were anonymized in accordance with confidentiality guidelines. This study was supported by Soonchunhyang University Research Fund.

### Data source and study population

This retrospective cohort study used data from the Korean National Health Insurance Service (NHIS), which provides mandatory health coverage to more than 97% of the Korean population. Data were accessed for research purposes in 01/07/2025. Because imaging modalities are not included in the NHIS health screening program, hepatic steatosis was assessed using the fatty liver index (FLI), a validated surrogate marker ranging from 0 to 100; values < 30 indicate low risk, whereas values ≥ 60 indicate high risk of SLD [11].

We initially identified 8,233,669 adults aged 20–79 years who participated in the 2017 National Health Screening Program. We excluded individuals with a prior diagnosis of HCC before the index year (International Classification of Diseases, 10th revision [ICD-10] code C22.0; n = 9,129), those who died within 1 year of screening (n = 5,264), those with insufficient diagnostic records to meet the operational definition of HCC (fewer than three HCC-related claims between 2018 and 2023; n = 1), those with incomplete health screening data (n = 27), those with documented chronic hepatitis B or C or HIV infection (ICD-10 codes B18.0–B18.2 and B20–B24; n = 258,173), and those with FLI < 60 (n = 6,719,515). The final analytic cohort comprised 1,241,560 individuals with FLI ≥ 60 (Supplementary Materials; Fig S1 in **S1 File**).

### Study outcome

The primary outcome was the development of HCC between January 1, 2018, and December 31, 2023. HCC was defined as three or more claims with the ICD-10 code C22.0, a claims-based definition previously validated in NHIS studies with a positive predictive value exceeding 90% [12]. The study population was followed from baseline until HCC diagnosis, death, or December 31, 2023, whichever occurred first.

### Variables

Baseline demographic and clinical characteristics were obtained from the 2017 health screening data. Demographic variables included age and sex. Anthropometric measurements included body mass index (BMI), waist circumference, and systolic and diastolic blood pressure. Laboratory parameters comprised fasting plasma glucose, alanine aminotransferase (ALT), aspartate aminotransferase (AST), gamma-glutamyl transferase (GGT), total cholesterol, low-density lipoprotein (LDL) cholesterol, high-density lipoprotein (HDL) cholesterol, triglycerides, hemoglobin, serum creatinine, estimated glomerular filtration rate (eGFR), and urine protein. Comorbidity was assessed using the Charlson Comorbidity Index (CCI), calculated from diagnostic codes recorded in the year preceding the index examination. ICD-10 codes are listed in Supplementary Materials; Table S1 in **S1 File**.

Lifestyle information was obtained from standardized self-administered questionnaires. Smoking status was categorized as never, former, or current. Alcohol consumption was categorized as none/mild or moderate-to-heavy. Based on the national screening criteria, moderate-to-heavy drinking was defined as consuming more than 7 units for men and 5 units for women at least twice weekly. Mild (social) drinking was defined as consuming up to 6 units for men and 4 units for women no more than once weekly. Physical activity was assessed as the self-reported number of weekly sessions of moderate-to-vigorous exercise.

Prior to model development, we performed data preprocessing to ensure data quality. Individuals with missing values in key variables were excluded (n = 27, corresponding to those with incomplete health screening data shown in the Fig. S1 in **S1 File**), and implausible values (e.g., physiologically impossible or extreme values) were removed based on predefined clinical criteria. For variables with limited missingness such as family history, missing values were treated as absence based on the structure of the questionnaire.

### Model development and selection

The study cohort was randomly divided into a development set (70%) and an internal validation set (30%), stratified by HCC status to maintain similar event rates in both sets. The development set was used for model training, hyperparameter tuning, and model selection, while the validation set was reserved for final assessment of model discrimination, calibration, and risk stratification.

To address extreme class imbalance (event rate 0.17%), we initially constructed a balanced training dataset including all HCC cases in the development set and a random sample of non-HCC controls at a 1:3 case-to-control ratio. On this subset, we trained and compared multiple predictive models. Deep learning architectures included standard deep neural networks (DNN), convolutional neural networks (CNN) for tabular data, a multi-head attention (MHA)–based DNN, and an MHA-CNN model. Classical ML algorithms included extreme gradient boosting (XGBoost), random forest (RF), gradient boosting machines (GBM), logistic regression (LR), support vector machines (SVM), naive Bayes (NB), and k-nearest neighbors (KNN). We also evaluated a hybrid ensemble model combining XGBoost and RF (Ensemble XGB–RF) to assess the potential benefit of aggregating tree-based methods. Hyperparameters were optimized using cross-validation. Deep learning models were trained with binary cross-entropy loss and the Adam optimizer, with early stopping to prevent overfitting.

Based on area under the receiver operating characteristic curve (AUROC) and F-measure in the validation set, the MHA-DNN, MHA-CNN, and XGBoost models were selected as the top three performers. MHA-DNN is a deep neural network architecture incorporating multi-head attention mechanisms, which enable the model to capture complex and non-linear relationships among input features by attending to multiple representation subspaces simultaneously [13]. MHA-CNN combines convolutional neural networks with multi-head attention to capture both local feature interactions and global dependencies [14]. XGBoost is an optimized gradient boosting algorithm based on decision trees, known for its efficiency and strong performance in structured data [15].

### Explainability and variable selection

To identify robust predictors of HCC, SHapley Additive exPlanations (SHAP) were applied to the top three models. Mean absolute SHAP values were calculated for each feature to quantify its global contribution to predictions. Common important variables, features consistently ranked highly across all three models, included AST, GGT, triglycerides, CCI, and FLI. To assess clinical and biological plausibility, these AI-selected features were further examined using Cox proportional hazards models and Kaplan–Meier survival analyses.

### Construction of the final MHA-DNN ensemble

MHA-DNN was chosen as the final base learner for risk prediction. We applied a repeated non-replacement undersampling strategy to generate 20 distinct training subsets, each containing all HCC cases and a unique set of non-HCC controls at a 1:3 ratio. An ensemble risk score was then constructed by aggregating predictions from the three best-performing MHA-DNN models, weighted at 0.7, 0.2, and 0.1, respectively.

### Performance evaluation and risk stratification

Model performance was assessed using AUROC, sensitivity, specificity, and F-measure at the optimal cutoff determined by the Youden index. For risk stratification and survival analysis, predicted risk scores were applied to the full dataset without sampling in both the training and validation cohorts. Finally, individuals in both the training and validation cohorts were categorized into four risk groups. Survival outcomes across these groups were compared using Kaplan–Meier curves and log-rank tests, and hazard ratios (HRs) were estimated using Cox proportional hazards models.

### Statistical analysis

Continuous variables are presented as means ± standard deviations and compared using Student’s t-test. Categorical variables are expressed as counts and percentages and compared using Chi-square tests. All analyses were conducted using SAS version 9.4 (SAS Institute, Cary, NC, United States) and R version 4.5.1 (R Foundation for Statistical Computing, Vienna, Austria; http://www.R-project.org). A two-sided *P*-value < 0.05 was considered statistically significant.

## Results

### Study population characteristics

Among 1,241,560 participants with FLI ≥ 60, 2,152 (0.17%) developed HCC over 6 years. Individuals who developed HCC were older, more frequently male, and had a higher prevalence of cirrhosis, diabetes, hypertension, and dyslipidemia compared with those who remained HCC-free (**Table 1**). Metabolic and biochemical profiles also differed between groups. Compared with individuals who did not develop HCC, those who developed HCC exhibited more adverse glycemic and liver enzyme profiles, while triglyceride and total cholesterol levels tended to be lower, despite a similar or higher burden of SLD (mean FLI, 78.3 ± 11.0 vs. 76.5 ± 11.0, *P* < 0.0001). Comorbidity burden, reflected by the CCI, was higher in the HCC group, consistent with clustering of metabolic and systemic risk factors.

**Table 1:** Baseline characteristics of study population stratified by hepatocellular carcinoma development.

Variables	Non-HCC (n = 1,239,408)	HCC (n = 2,152)	Total (n = 1,241,560)	*P*-value
Age (years)	41.5 ± 12.4	57.0 ± 10.5	41.5 ± 12.4	<.0001
Male	942,023 (76.0)	1,757 (81.6)	943,780 (76.0)	<.0001
Cirrhosis (%)	4,894 (0.4)	423 (19.7)	5,317 (0.4)	<.0001
Diabetes (%)	125,625 (10.1)	887 (41.2)	126,512 (10.2)	<.0001
Hypertension (%)	275,651 (22.2)	1,178 (54.7)	276,829 (22.3)	<.0001
Dyslipidemia (%)	207,132 (16.7)	529 (24.6)	207,661 (16.7)	<.0001
Number of exercise/week (%)				<.0001
None	504,554 (40.7)	1,134 (52.7)	505,688 (40.7)	
1–2/week	418,102 (33.7)	446 (20.7)	418,548 (33.7)	
3–4/week	204,813 (16.5)	320 (14.9)	205,133 (16.5)	
≥ 5/week	111,939 (9.0)	252 (11.7)	112,191 (9.0)	
Fatty liver index	76.5 ± 11.0	78.3 ± 11.0	76.5 ± 11.0	<.0001
Charlson comorbidity index	2.5 ± 2.3	5.4 ± 3.2	2.5 ± 2.3	<.0001
Smoking				<.0001
Non-smoker	465,702 (37.6)	803 (37.3)	466,505 (37.6)	
Ex-smoker	269,287 (21.7)	598 (27.8)	269,885 (21.7)	
Current smoker	504,419 (40.7)	751 (34.9)	505,170 (40.7)	
Alcohol				<.0001
None or mild	916,635 (74.0)	1,641 (76.3)	918,276 (74.0)	
Moderate to heavy†	322,773 (26.0)	511 (23.7)	323,284 (26.0)	
BMI (kg/m^2^)	26.3 ± 3.8	25.3 ± 3.6	26.3 ± 3.8	<.0001
AST (U/L)	32.6 ± 30.8	49.3 ± 37.5	32.6 ± 30.8	<.0001
ALT (U/L)	40.3 ± 34.7	39.4 ± 29.9	40.3 ± 34.7	1
GGT (U/L)	65.6 ± 76.8	161.7 ± 210.2	65.8 ± 77.4	<.0001
Fasting glucose (mg/dL)	107.5 ± 32.0	123.1 ± 43.3	107.5 ± 32.0	<.0001
Total cholesterol (mg/dL)	208.1 ± 42.4	175.7 ± 41.7	208.1 ± 42.4	<.0001
Triglycerides (mg/dL)	247.8 ± 176.2	177.6 ± 151.0	247.7 ± 176.2	<.0001
HDL cholesterol (mg/dL)	49.3 ± 21.7	50.5 ± 17.1	49.3 ± 21.7	<.0001
LDL cholesterol (mg/dL)	114.5 ± 39.6	92.3 ± 35.9	114.4 ± 39.6	<.0001
eGFR (mL/min/1.73m^2^)	93.5 ± 37.1	86.6 ± 24.8	93.5 ± 37.0	<.0001

Data are presented as means ± standard deviations for continuous variables and n (%) for categorical variables.

ALT, alanine aminotransferase; AST, aspartate aminotransferase; BMI, body mass index; eGFR, estimated glomerular filtration rate; GGT, gamma-glutamyl transferase; HCC, hepatocellular carcinoma; HDL, high-density lipoprotein; LDL, low-density lipoprotein.

^†^≥ 2 occasions per week, with ≥ 7 drinks per occasion for men and ≥ 5 drinks per occasion for women.

### Performance of candidate models

**Fig 1:**
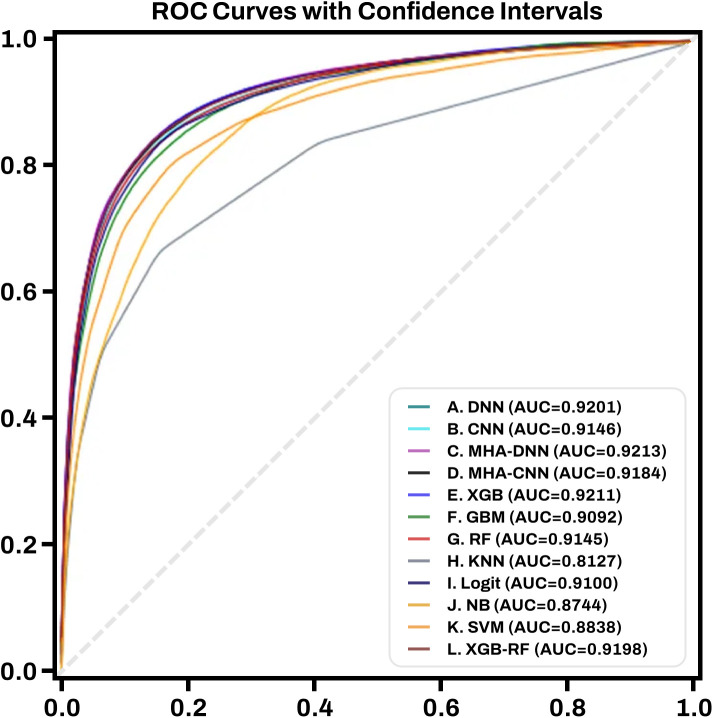
Receiver operating characteristic curves of 12 artificial intelligence algorithms for predicting hepatocellular carcinoma in steatotic liver disease.

To identify the most effective approach for predicting HCC, we first evaluated the performance of various ML and deep learning architectures (Supplementary Materials; Table S2 in **S1 File**). The MHA-DNN, MHA-CNN, and XGBoost achieved the highest validation AUROC values (~0.92) along with favorable sensitivity, specificity, F-measure, and geometric mean (**Fig 1**). Other models, such as RF and LR, showed slightly lower sensitivity or overall F-measure at comparable specificity. Based on these results, the MHA-DNN, MHA-CNN, and XGBoost were selected as the top-performing models for SHAP-based interpretation and variable selection.

### Identification of common key predictors via explainable AI

**Fig 2:**
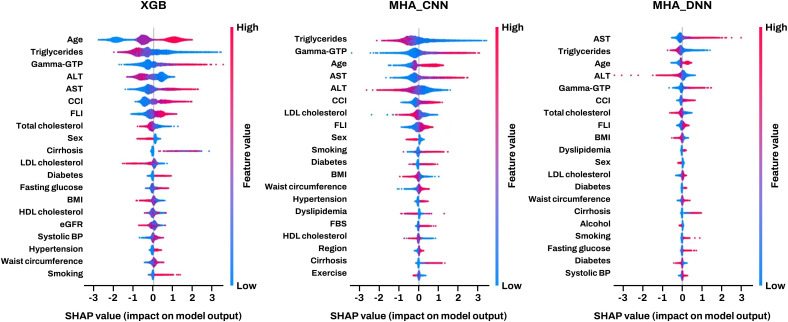
SHAP-based feature importance for the three top-performing models. Summary plot showing the impact of each feature on hepatocellular carcinoma risk predictions. Features are ranked by mean absolute SHAP values across all predictions. The color gradient represents feature values (red = high, blue = low). Horizontal position indicates the effect on model output, with positive values increasing HCC prediction probability. Age was the most important feature, followed by gamma-glutamyl transferase and liver transaminases. The consistent rankings across different model architectures support the biological relevance of these predictors.

SHAP analysis of the top three models revealed a consistent set of important predictors, including age, sex, triglycerides, total cholesterol, AST, ALT, GGT, CCI, and FLI. SHAP summary plots (**Fig 2**) provided a granular view of how these features influenced model output; notably, lower values of TG and total cholesterol were frequently associated with an increased HCC risk.

Kaplan–Meier analyses further demonstrated distinct risk patterns for incident HCC across categories of these predictors. For instance, age and liver enzymes showed clear dose-dependent associations. For triglyceride levels (Supplementary Materials; Fig. S2G in **S1 File**), the group with triglycerides < 150 mg/dL exhibited a significantly higher cumulative incidence of HCC compared to the group with triglycerides ≥ 150 mg/dL (P < 0.0001), supporting the inverse association captured by the models.

Using the common important variables, we performed explanatory Cox regression analyses (**Table 2**). Because ALT was strongly correlated with AST (Supplementary Materials; Fig S3 in **S1 File**), only AST was included in multivariable Cox models to avoid multicollinearity. All eight variables, including older age, male sex, elevated GGT, higher AST, and lower lipid levels, showed significant associations with incident HCC in univariate analyses and remained independently associated after adjustment (**Table 2**).

**Table 2:** High-risk features for hepatocellular carcinoma: univariable and multivariable hazard ratios across model rankings.

Risk Factor	No HCC cases (%)	HCC cases (%)	Univariate		Multivariate*		Model rank		
			HR (95% CI)	*P*-value	HR (95% CI)	*P*-value	XGB	MHA_CNN	MHA_DNN
Age, years							1st	3rd	3rd
20 ~ 39	407,840 (32.9)	54 (2.5)	1 (Ref.)	–	1 (Ref.)	–			
40 ~ 59	584,952 (47.2)	532 (24.7)	6.89 (5.21–9.12)	<.0001	5.92 (4.46–7.86)	<.0001			
60 ~ 79	246,616 (19.9)	1,566 (72.8)	49.2	<.0001	34.04	<.0001			
			(37.51–64.54)		(25.75–44.98)				
Sex							9th	9th	11th
Male	942,023 (76.0)	1,757 (81.6)	1.40 (1.26–1.56)	<.0001	2.16 (1.93–2.42)	<.0001			
Female	297,385 (24.0)	395 (18.4)	1 (Ref.)	–	1 (Ref.)	–			
FLI							7th	8th	8th
< 80	757,033 (61.1)	1,165 (54.1)	1 (Ref.)	–	1 (Ref.)	–			
≥ 80	482,375 (38.9)	987 (45.9)	1.33 (1.22–1.45)	<.0001	1.65 (1.52–1.80)	<.0001			
CCI							6th	6th	6th
0	215,871 (17.4)	85 (3.9)	1 (Ref.)	–	1 (Ref.)	–			
1	288,302 (23.3)	131 (6.1)	1.15 (0.88–1.52)	0.31	0.91 (0.69–1.20)	0.50			
2	249,934 (20.2)	220 (10.2)	2.24 (1.74–2.87)	<.0001	1.27 (0.99–1.64)	0.06			
≥ 3	485,301 (39.2)	1,716 (79.7)	9.08 (7.30–11.28)	<.0001	2.49 (1.99–3.11)	<.0001			
AST							5th	4th	1st
< 40	1,002,640 (80.9)	1,127 (52.4)	1 (Ref.)	–	1 (Ref.)	–			
≥ 40	236,768 (19.1)	1,025 (47.6)	3.85 (3.54–4.19)	<.0001	2.13 (1.94–2.34)	<.0001			
ALT							4th	5th	4^th^
< 40	777,026 (62.7)	1,384 (64.3)	1 (Ref.)	–	–	–			
≥ 40	462,382 (37.3)	768 (35.7)	0.93 (0.85–1.02)	0.10	–	–			
GGT							3rd	2nd	5th
< 70	891,761 (72)	1,019 (47.4)	1 (Ref.)	–	1 (Ref.)	–			
≥ 70	347,647 (28)	1,133 (52.6)	2.85 (2.62–3.10)	<.0001	2.94 (2.67–3.23)	<.0001			
Total cholesterol							8^th^	21st	7th
< 200	536,791 (43.3)	1,594 (74.1)	1 (Ref.)	–	1 (Ref.)	–			
≥ 200	702,617 (56.7)	558 (25.9)	0.27 (0.24–0.29)	<.0001	0.47 (0.42–0.52)	<.0001			
Triglyceride							2nd	1st	2^nd^
< 150	313,622 (25.3)	1,204 (55.9)	1 (Ref.)	–	1 (Ref.)	–			
≥ 150	925,786 (74.7)	948 (44.1)	0.27 (0.25–0.29)	<.0001	0.26 (0.24–0.29)	<.0001			

*Adjusted for age, sex, triglycerides, total cholesterol, gamma-glutamyl transferase, aspartate aminotransferase, fatty liver index, and Charlson Comorbidity Index.

ALT, alanine aminotransferase; AST, aspartate aminotransferase; CCI, Charlson Comorbidity Index; FLI, fatty liver index; GGT, gamma-glutamyl transferase; HR, hazard ratio; MHA_CNN, multi-head attention convolutional neural network; MHA_DNN, multi-head attention deep neural network; TG, triglycerides; XGB, extreme gradient boosting.

### Development and performance of the final weighted ensemble model

Given the superior performance of the MHA-DNN, we developed a final weighted voting ensemble combining predictions from the three best-performing iterations among 20 repeated non-replacement undersampling training sessions. In the independent validation set, this ensemble model achieved an AUROC of 0.9237, sensitivity of 71.36%, and specificity of 93.65% (**Table 3**), comparable to performance in the training set (AUROC 0.9277), suggesting minimal overfitting.

**Table 3:** Performance of the final multi-head attention deep neural network ensemble.

Metric	Development cohort	Internal validation cohort
Accuracy	0.8858	0.8808
Sensitivity (recall)	0.7278	0.7136
Specificity	0.9385	0.9365
AUROC	0.9277	0.9237
F measure	0.7611	0.7496
Geometric mean	0.8264	0.8175

AUROC, area under the receiver operating characteristic curve.

**Fig 3:**
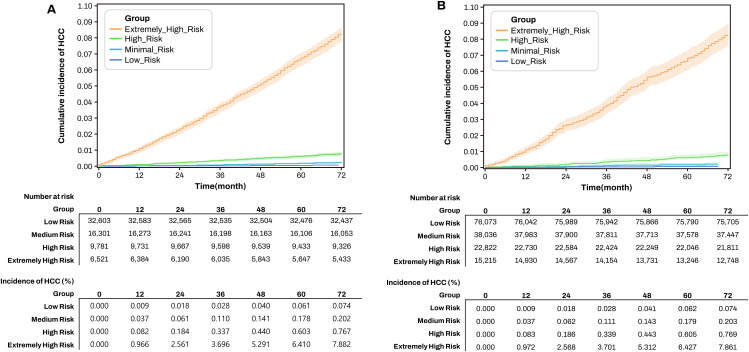
Kaplan–Meier curves for cumulative incidence of HCC according to predicted risk groups. **(A)** Validation cohort. **(B)** Training cohort.

The population was stratified into four risk groups: low, medium, high, and extremely high risk. Kaplan–Meier analysis based on predicted risk scores demonstrated clear separation of HCC-free survival across risk groups in both the validation cohort (**Fig 3A**) and the training cohort (**Fig 3B**) (*P* < 0.0001 for both). The cumulative incidence of HCC increased stepwise across risk groups, with the highest incidence observed in the extremely high-risk group (Fig 3). Similar patterns were observed in both cohorts, supporting the robustness and consistency of the model. In univariate Cox models, HRs for HCC compared with the low-risk group were 7.0 for medium-risk, 37.5 for high-risk, and 74.9 for extremely high-risk individuals. The cumulative incidence in the extremely high-risk group approached levels typically used as thresholds for HCC surveillance in patients with cirrhosis.

## Discussion

In this nationwide cohort of over 1.2 million adults with SLD, we developed and validated an explainable ML model to identify individuals with SLD at increased risk of HCC. In the internal validation cohort, the final model demonstrated high discrimination (AUROC 0.9237) and effectively stratified the population into risk groups with markedly different cumulative incidences. Although the extremely high-risk stratum represented only a small fraction of the cohort, it had an approximately 75-fold higher hazard of HCC than the low-risk group.

Model interpretation suggested a biologically coherent metabolic phenotype associated with HCC risk. SHAP analysis revealed a ‘metabolic burnout’ signature, characterized by lower levels of triglycerides and total cholesterol alongside elevated AST and GGT in individuals at high risk for HCC. This pattern is consistent with prior observations of impaired hepatic lipoprotein export and metabolic derangement in advanced fibrosis and burned-out steatohepatitis [16,17].

Specifically, the high predictive value of GGT and AST may reflect persistent hepatic injury, oxidative stress, and systemic inflammation, which have been implicated in hepatocarcinogenesis [18,19]. Furthermore, our findings are consistent with prior reports that progression toward advanced fibrosis may be accompanied by lower lipid levels [20,21]. While categorical analysis in the Kaplan–Meier curves (Fig. S2G in **S1 File**) primarily highlights this inverse relationship, our MHA-based models incorporated these values as continuous multivariable features within a broader risk-prediction framework. The superior performance of MHA-based models may reflect their ability to integrate these metabolic signatures with systemic factors, such as the CCI, which captures the cumulative burden of chronic comorbidity.

Methodologically, we addressed extreme class imbalance using repeated non-replacement undersampling and ensembling. We conducted 20 independent training sessions with non-replacement undersampling and constructed a final weighted voting MHA-DNN ensemble by aggregating predictions from the three best-performing iterations. This approach improved stability in a rare-event setting (0.17% event rate) while preserving sensitivity for HCC detection without compromising specificity [22]. It also complements linear noninvasive fibrosis tests, such as FIB-4 and the NAFLD fibrosis score, which primarily identify advanced fibrosis rather than predict incident HCC [23].

These findings may inform refinement of surveillance strategies in SLD. Current international guidelines generally discourage universal HCC surveillance in individuals with SLD without cirrhosis [6,24]. In contrast, the extremely high-risk stratum identified by our model likely exceeds commonly cited annual incidence thresholds for surveillance cost-effectiveness [25,26]. If externally validated, this framework could support targeted surveillance for high-risk individuals while reducing unnecessary testing in low-risk populations.

Our study has several limitations. First, steatosis was defined using FLI rather than histology or transient elastography, although FLI is well validated for epidemiologic studies [11,27,28]. The retrospective design and reliance on administrative data may introduce misclassification despite rigorous exclusion criteria. Second, in the Korean NHIS system, patients with confirmed HCC are typically registered in a special reimbursement program that requires strict diagnostic criteria and is closely linked to ICD-10 code C22.0. Therefore, misclassification of typical HCC cases under alternative codes such as C22.8 is likely to be minimal, although a small degree of underestimation cannot be excluded. Third, the cohort was exclusively Korean, requiring external validation in diverse populations, particularly given ethnic differences in adiposity distribution and metabolic risk [29]. Finally, a simplified risk score based on conventional statistical models, such as Cox regression using selected predictors, may be feasible and could improve clinical usability. However, such approaches may not fully capture the complex non-linear interactions identified by machine learning models. Future studies should focus on developing and validating simplified scoring systems derived from these findings.

In conclusion, an explainable ML model effectively stratified HCC risk in a large SLD population, highlighting a metabolic burnout signature of low triglycerides and liver injury. This approach may provide a basis for future external validation and evaluation of risk-based surveillance strategies.

## Supporting information

S1 File Supplementary materials. Supplementary figures and tables including Fig. S1–S3 and Tables S1–S2.
(DOCX)

## Abbreviations

ALT: alanine aminotransferase
AST: aspartate aminotransferase
AUROC: area under the receiver operating characteristic curve
BMI: body mass index
CCI: Charlson comorbidity index
CNN: convolutional neural network
DNN: deep neural network
eGFR: estimated glomerular filtration rate
FLI: fatty liver index
GBM: gradient boosting machine
GGT: gamma-glutamyl transferase
HCC: hepatocellular carcinoma
HDL: high-density lipoprotein
HRs: hazard ratios
ICD-10: International Classification Of Diseases, 10th revision
IRB: Institutional Review Board
KNN: k-nearest neighbors
LDL: low-density lipoprotein
LR: logistic regression
MHA: multi-head attention
ML: machine learning
NB: naive Bayes
NHIS: National Health Insurance Service
RF: random forest
SHAP: Shapley additive explanations
SLD: steatotic liver disease
SVM: support vector machine
XGBoost: extreme gradient boosting
