# Supplementary material for: Machine learning-based prediction of hepatocellular carcinoma risk in steatotic liver disease: A nationwide cohort study
Source: PLoS One. 2026 May 28;21(5):e0349593. doi: 10.1371/journal.pone.0349593 (PMC13218536; doi:10.1371/journal.pone.0349593)
Supplement: S1 File — Supplementary figures and tables including Fig. S1–S3 and Tables S1–S2. (DOCX) [file pone.0349593.s001.docx]

**SUPPLEMENTARY MATERIALS**

**Study Title**

Machine learning-based prediction of hepatocellular carcinoma risk in steatotic liver disease: A nationwide cohort study

**Authors**

Log Young Kim, Ji Soo Lee, Jeong-Ju Yoo, Eun Ju Cho, Sang Gyune Kim, Young Seok Kim

**Table of Contents**

[Table S1. Charlson Comorbidity Index definition based on ICD-10 codes 2](#_Toc218459759)

[Table S2. Performance of various models for predicting hepatocellular carcinoma in steatotic liver disease 4](#_Toc218459760)

[Fig. S1. Flowchart of study population 6](#_Toc218459761)

[Fig. S2. Kaplan–Meier curves for incident hepatocellular carcinoma according to categories of the eight key risk factors. 7](#_Toc218459762)

[Fig. S3. Correlation matrix of candidate predictors for incident hepatocellular carcinoma in steatotic liver disease 8](#_Toc218459763)

# **Table S1. Charlson Comorbidity Index definition based on ICD-10 codes**

| **Comorbidity** | **ICD-10 code** |
| --- | --- |
| Hypertension | I10 I11 I12 I13 I15 |
| Myocardial infarction | I21 I22 I252 |
| Congestive heart failure | I43 I50 I099 I110 I130 I132 I255 I420 I425 I426 'I427 I428 I429 P290 |
| Peripheral vascular disease | I70 I71 I731 I738 I739 I771 I790 I792 K551 K558 K559 Z958 Z959 |
| Cerebrovascular disease | G45 G46 I60 I61 I62 I63 I64 I65 I66 I67 I68 I69 H340 |
| Dementia | F00 F01 F02 F03 G30 G311 F051 |
| Chronic pulmonary disease | J40 J41 J42 J43 J44 J45 J46 J47 J60 J61 J62 J63 J64 J65 J66 J67 I278 I279 J684 J701 J703 |
| Rheumatic disease | M05 M06 M32 M33 M34 M315 M351 M353 M360 |
| Peptic ulcer disease | K25 K26 K27 K28 |
| Mild liver disease | B18 K73 K74 K700 K701 K702 K703 K709 K713 K714 K715 K717 K760 K762 K763 K764 K768 K769 Z944 |
| Diabetes without chronic complication | E100 E101 E106 E108 E109 E110 E111 E116 E118 E119 E120 E121 E126 E128 E129 E130 E131 E136 E138 E139 E140 E141 E146 E148 E149 |
| Diabetes with chronic complication | E102 E103 E104 E105 E107 E112 E113 E114 E115 E117 E122 E123 E124 E125 E127 E132 E133 E134 E135 E137 E142 E143 E144 E145 E147 |
| Hemiplegia or paraplegia | G81 G82 G041 G114 G801 G802 G830 G831 G832 G833 G834 G839 |
| Renal disease | N18 N19 I120 I131 N032 N033 N034 N035 N036 N037 N052 N053 N054 N055 N056 N057 N250 Z490 Z491 Z492 Z940 Z992 |
| Any malignancy | C00 C01 C02 C03 C04 C05 C06 C07 C08 C09 C10 C11 C12 C13 C14 C15 C16 C17 C18 C19 C20 C21 C22 C23 C24 C25 C26 C30 C31 C32 C33 C34 C37 C38 C39 C40 C41 C43 C45 C46 C47 C48 C49 C50 C51 C52 C53 C54 C55 C56 C57 C58 C60 C61 C62 C63 C64 C65 C66 C67 C68 C69 C70 C71 C72 C73 C74 C75 C76 C81 C82 C83 C84 C85 C88 C90 C91 C92 C93 C94 C95 C96 C97 |
| Moderate or severe liver disease | I850 I859 I864 I982 K704 K711 K721 K729 K765 K766 K767 |
| Metastatic solid tumor | C77 C78 C79 C80 |
| Thyroid dysfunction | E00 E01 E02 E03 E04 E05 E06 E07 |
| Renal failure | N17 N18 N19 |

# **Table S2. Performance of various models for predicting hepatocellular carcinoma in steatotic liver disease**

|  | **Accuracy** | **Recall (TPR)** | **Specificity (TNR)** | **AUC** | **F-measure** | **G-mean** |
| --- | --- | --- | --- | --- | --- | --- |
| **k-nearest neighbors** | 0.8305 [0.8201, 0.8415] | 0.5097 [0.4830, 0.5432] | 0.9375 [0.9272, 0.9472] | 0.8127 [0.7966, 0.8297] | 0.6004 [0.5708, 0.6273] | 0.6911 [0.6719, 0.7138] |
| **Logistic regression** | 0.8743 [0.8637, 0.8848] | 0.6676 [0.6317, 0.6980] | 0.9434 [0.9342, 0.9528] | 0.9100 [0.8978, 0.9196] | 0.7266 [0.7041, 0.7485] | 0.7935 [0.7722, 0.8117] |
| **Naïve Bayes** | 0.8269 [0.8128, 0.8403] | 0.6324 [0.5830, 0.6826] | 0.8918 [0.8712, 0.9134] | 0.8744 [0.8615, 0.8859] | 0.6458 [0.6130, 0.6719] | 0.7507 [0.7226, 0.7771] |
| **Support vector machine** | 0.8526 [0.8405, 0.8616] | 0.5664 [0.5369, 0.5993] | 0.9479 [0.9364, 0.9557] | 0.8838 [0.8691, 0.8961] | 0.6573 [0.6307, 0.6844] | 0.7326 [0.7133, 0.7536] |
| **Random Forest** | 0.8775 [0.8681, 0.8875] | 0.6676 [0.6373, 0.6983] | 0.9474 [0.9355, 0.9585] | 0.9145 [0.9048, 0.9239] | 0.7312 [0.7084, 0.7522] | 0.7952 [0.7765, 0.8121] |
| **XGBoost** | 0.8811 [0.8701, 0.8918] | 0.6998 [0.6677, 0.7299] | 0.9419 [0.9311, 0.9513] | 0.9211 [0.9109, 0.9306] | 0.7470 [0.7214, 0.7691] | 0.8118 [0.7923, 0.8276] |
| **GBM** | 0.8660 [0.8550, 0.8783] | 0.6027 [0.5701, 0.6354] | 0.9538 [0.9439, 0.9617] | 0.9092 [0.8971, 0.9199] | 0.6921 [0.6633, 0.7189] | 0.7581 [0.7381, 0.7780] |
| **Enssemble XGB-RF** | 0.8811 [0.8691, 0.8910] | 0.6917 [0.6569, 0.7228] | 0.9445 [0.9349, 0.9581] | 0.9198 [0.9088, 0.9279] | 0.7444 [0.7186, 0.7657] | 0.8082 [0.7876, 0.8264] |
| **DNN** | 0.8855, [0.8753, 0.8941] | 0.7185, [0.6752, 0.7650] | 0.9413, [0.9258, 0.9575] | 0.9201, [0.9082, 0.9307] | 0.7584, [0.7376, 0.7795] | 0.8222, [0.7983, 0.8433] |
| **CNN** | 0.8805, [0.8711, 0.8899] | 0.7017, [0.6466, 0.7530] | 0.9405, [0.9203, 0.9553] | 0.9146, [0.9046, 0.9268] | 0.7467, [0.7240, 0.7676] | 0.8121, [0.7843, 0.8374] |
| **MHA_DNN** | 0.8861, [0.8736, 0.8939] | 0.7139, [0.6582, 0.7683] | 0.9435, [0.9257, 0.9584] | 0.9213, [0.9119, 0.9323] | 0.7578, [0.7313, 0.7814] | 0.8204, [0.7928, 0.8446] |
| **MHA_CNN** | 0.8826, [0.8724, 0.8932] | 0.7035, [0.6366, 0.7726] | 0.9424, [0.9188, 0.9624] | 0.9184, [0.9084, 0.9279] | 0.7498, [0.7209, 0.7769] | 0.8138, [0.7801, 0.8447] |

# **Fig. S1. Flowchart of study population**

**
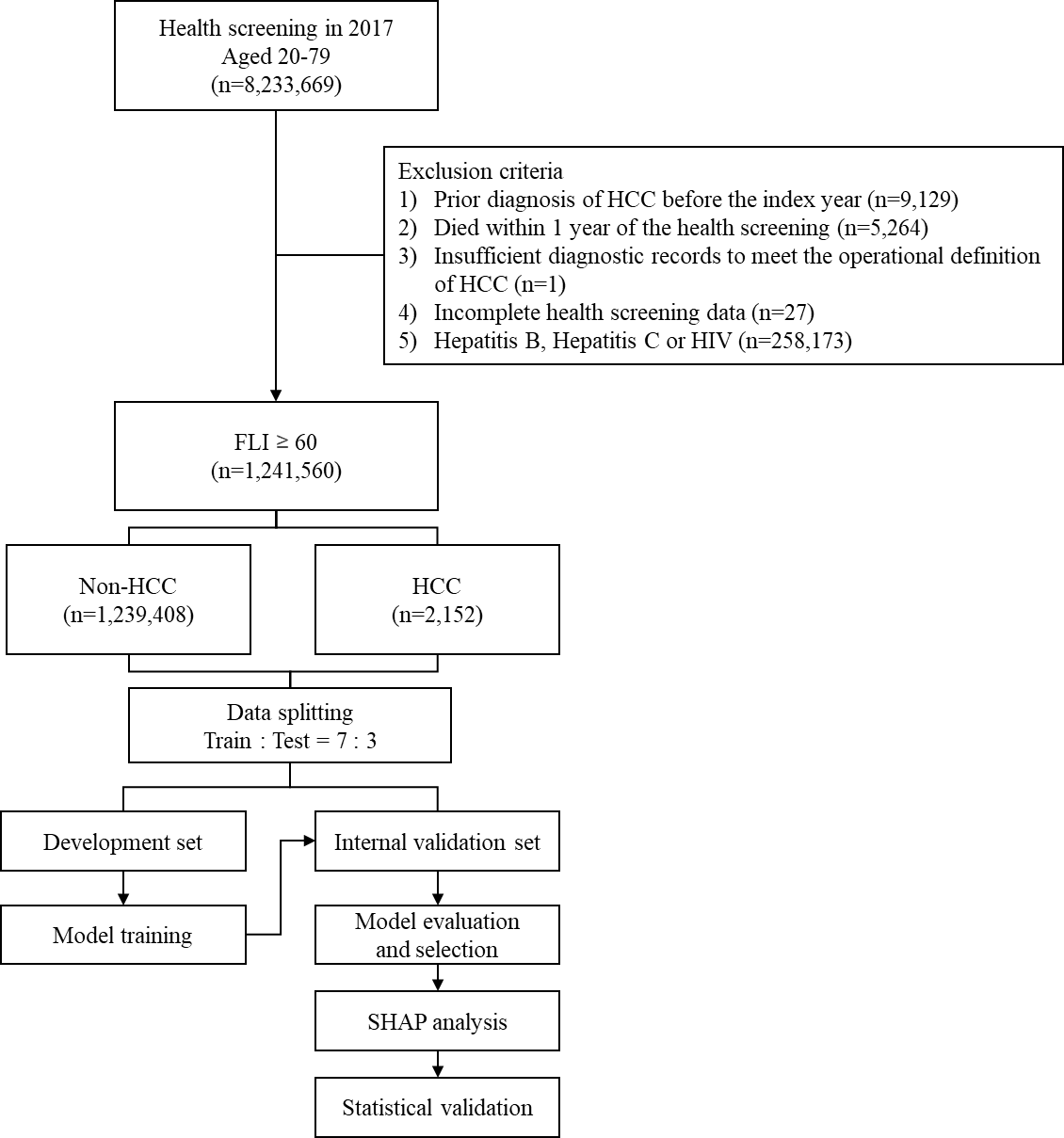
**

**Fig. S2. Kaplan–Meier curves for incident hepatocellular carcinoma according to categories of the eight key risk factors** (A) Age stratification showing pronounced risk separation above median age. (B) Gamma-glutamyl transferase levels demonstrating dose-dependent risk escalation. (C) Triglyceride levels revealing U-shaped relationship with HCC risk. (D) Fatty liver index categories showing progressive risk increase. All comparisons p<0.0001 by log-rank test. Shaded areas represent 95% confidence intervals.


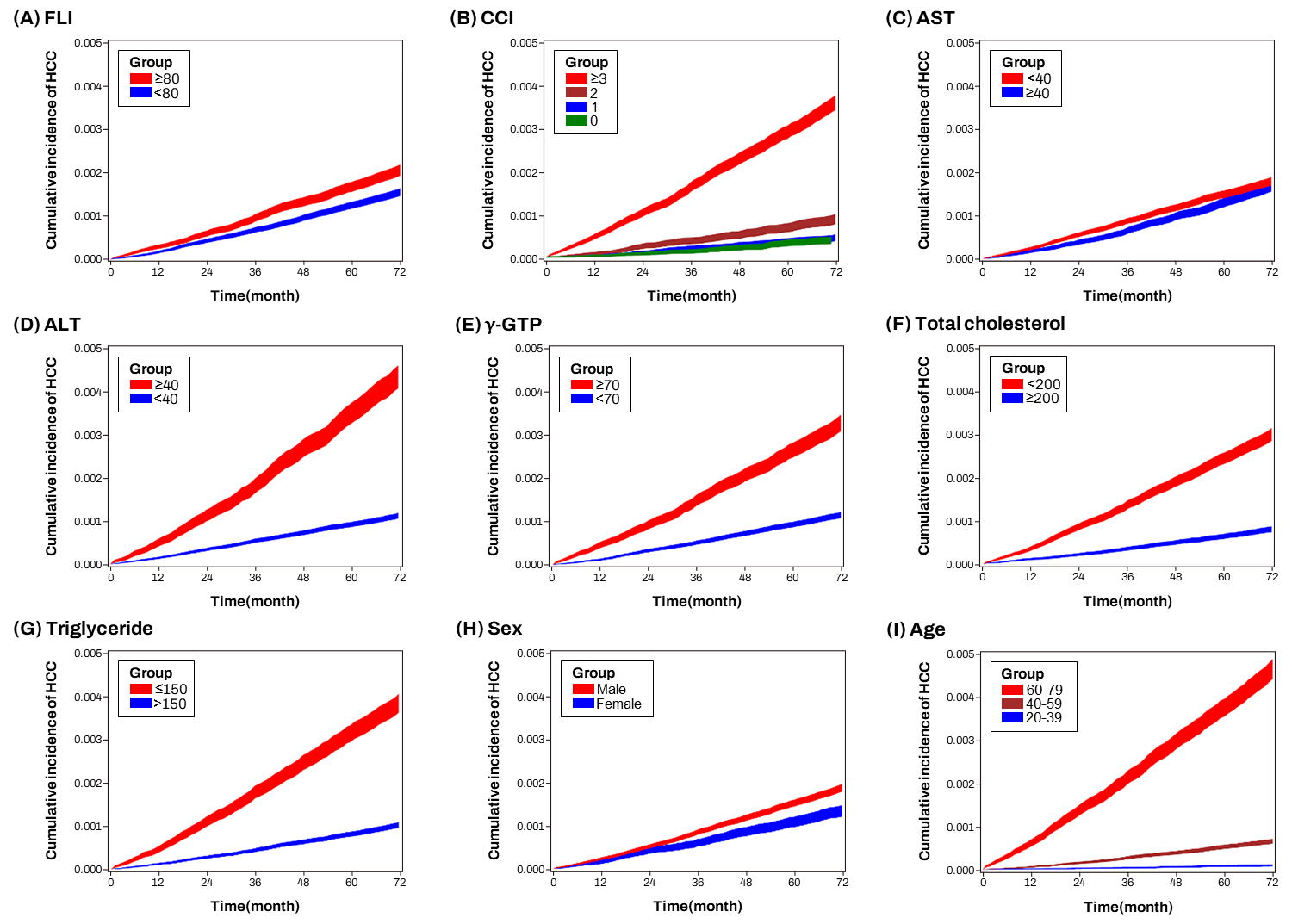


# **Fig. S3. Correlation matrix of candidate predictors for incident hepatocellular carcinoma in steatotic liver disease**

Correlation matrix of the 30 baseline candidate variables considered in the artificial intelligence models, including demographic, anthropometric, laboratory, comorbidity and lifestyle factors. Each cell represents the pairwise correlation coefficient between two variables, with color intensity indicating the strength and direction of the association. Variables comprise age, sex, body mass index, waist circumference, blood pressure, fasting glucose, triglycerides, high-density lipoprotein cholesterol, low-density lipoprotein cholesterol, total cholesterol, aspartate aminotransferase, alanine aminotransferase, gamma-glutamyl transferase, creatinine, estimated glomerular filtration rate, hemoglobin, proteinuria, Charlson Comorbidity Index, liver cirrhosis, diabetes mellitus, hypertension, dyslipidemia, smoking, alcohol consumption, physical activity, income level and fatty liver index. The matrix illustrates clusters of closely related features, such as correlations among lipid parameters and between liver enzymes, and informs model development and interpretation of feature importance.

**
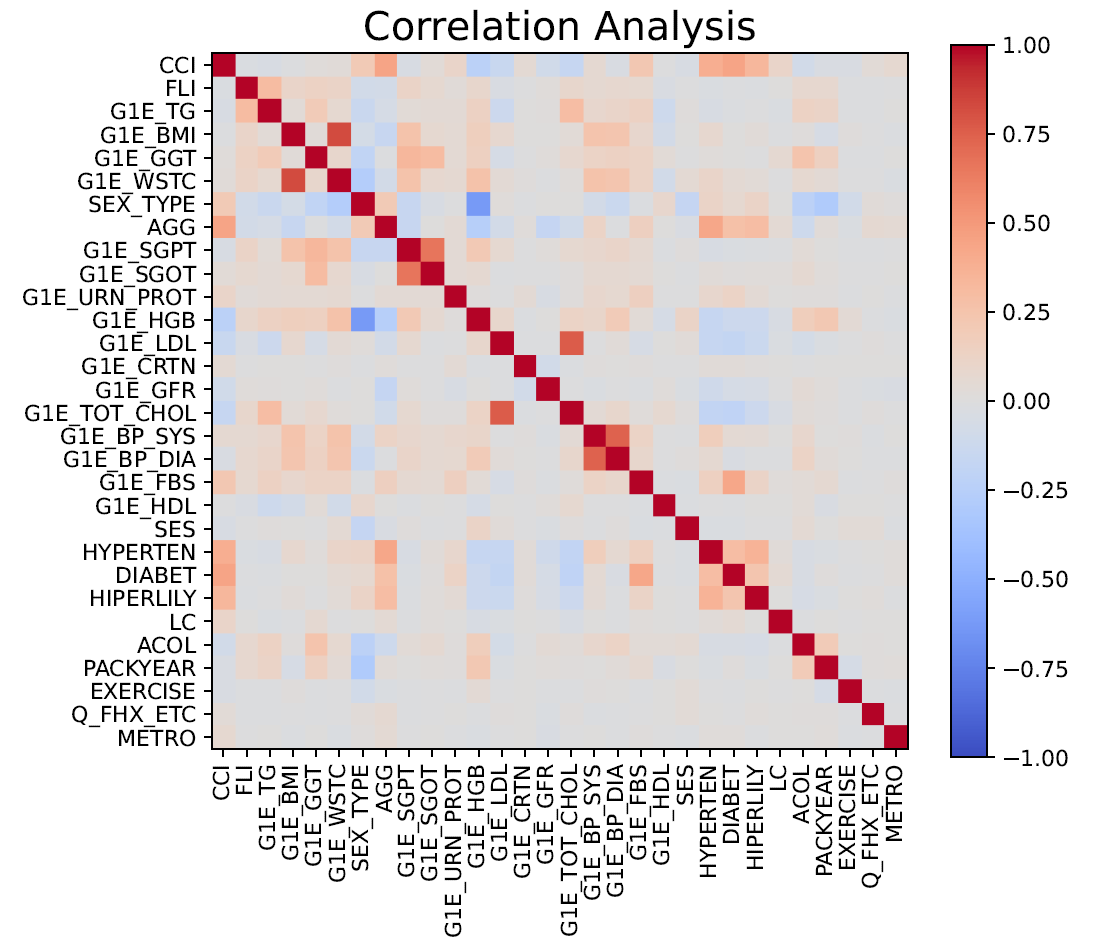
**
